# P-679. Risk Factors Shared Among Common Respiratory Viruses

**DOI:** 10.1093/ofid/ofae631.875

**Published:** 2025-01-29

**Authors:** Bennett Waxse, Tam Tran, Huan Mo, Joshua Denny

**Affiliations:** NIAID/CNH, Bethesda, Maryland; NHGRI, Bethesda, Maryland; NHGRI, Bethesda, Maryland; NHGRI, Bethesda, Maryland

## Abstract

**Background:**

Upper respiratory infections affect patients of all ages, with severity influenced by demographics, comorbidities, and immunosuppression. Studies often determine risk factors associated with severe disease, but rarely are multiple upper respiratory pathogens compared within the same cohort. Using electronic health record (EHR) phenotyping of 267,031 participants with relevant EHR data enrolled in the National Institute of Health’s All of Us Research Program (All of Us), we identified and validated cohorts of participants, and revealed shared risk factors for severity among upper respiratory viruses.

**Figure:**
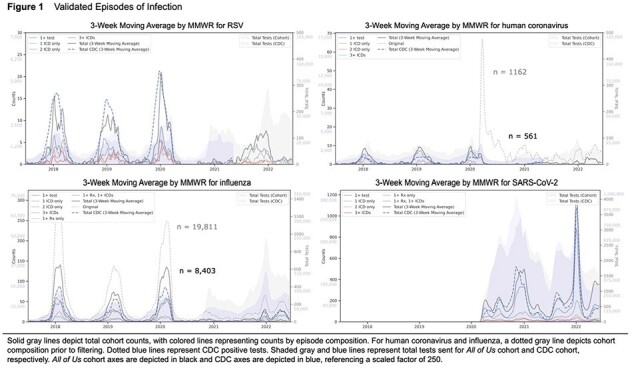

**Methods:**

We developed EHR phenotype algorithms for eight respiratory viruses using pathogen-specific billing codes, test results, and treatment-dose antivirals. We identified episodes of infection over 90-day periods and validated these using CDC national detection rates over the same period. We then used multivariable logistic regression to compare demographics and identify predictors of hospital admission, adjusting for age, sex, self-identified race and ethnicity, BMI, smoking status, and EHR record length.

**Figure:**
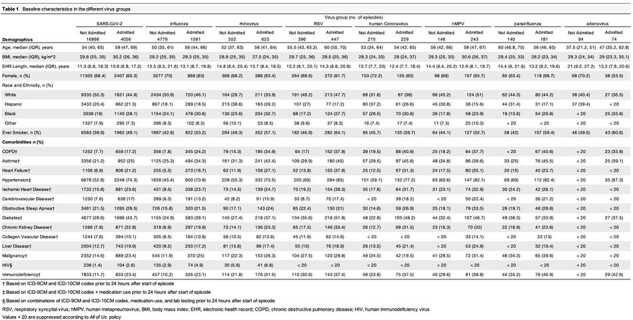

**Results:**

Scaled episode and testing counts paralleled CDC data, with expected seasonal trends observed for influenza, parainfluenza, respiratory syncytial virus (RSV), rhinovirus, human metapneumovirus (hMPV), SARS-CoV-2, and human coronavirus (hCOV) (RSV, hCOV, influenza, and SARS-CoV-2 shown in Figure 1). Across viruses, hospital admission was associated with older age, male sex, underweight, severe obesity, self-identified Black race, and smoking (Table 1). Notably, significant predictors of admission included COPD, asthma, heart failure, ischemic heart disease, hypertension, diabetes, chronic kidney disease, and immunodeficiency, even after adjusting for confounding variables (Figure 2).

**Figure:**
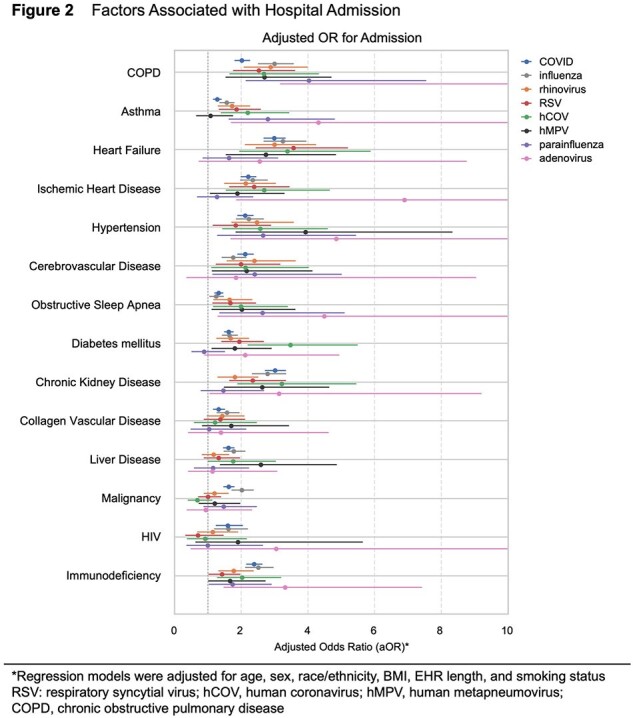

**Conclusion:**

In this study we established and validated an EHR phenotyping algorithm, and demonstrated that clinical risk factors — particularly cardiopulmonary, metabolic, and immunological comorbidities — are associated with severe outcomes among multiple upper respiratory viruses. These results suggest that universal prevention interventions may mitigate the impact of respiratory viral infections in high-risk populations.

**Disclosures:**

**All Authors**: No reported disclosures

